# Variation in facility-level likelihood of drug-resistant *Staphylococcus aureus* in outpatients remains after patient-level risk adjustment

**DOI:** 10.1017/ash.2025.10127

**Published:** 2025-09-10

**Authors:** Margaret Carrel, Qianyi Shi, Shinya Hasegawa, Christine Bricker, Miah Boyle, Michihiko Goto

**Affiliations:** 1 Department of Geographical & Sustainability Sciences, University of Iowa, Iowa City, IA, USA; 2 Department of Internal Medicine, University of Iowa, Iowa City, IA, USA; 3 Center for Access & Delivery Research and Evaluation (CADRE), Iowa City Veterans Affairs Health Care System, Iowa City, IA, USA; 4 Department of Political Science, University of Iowa, Iowa City, IA, USA

## Abstract

**Objective:**

Effective empiric therapy options for *Staphylococcus aureus* infections are limited by rising rates of resistance to non-beta lactam antimicrobial agents. The use of cumulative susceptibility testing results, antibiograms, are promoted as a tool for improving empiric therapy decisions, but it is unclear how much of the variation in antibiograms between facilities and the associated efficacy of antimicrobial agents is driven by underlying differences in patient characteristics such as comorbidities and prior antibiotic exposure.

**Design:**

Retrospective cohort study of 46,866 *S. aureus* specimens from outpatient settings of the Veterans Health Administration (VHA) from 2021 and 2022 linked to electronic health record information on patient comorbidities, prior antibiotic usage, age and gender.

**Setting:**

Outpatient VHA clinics in the conterminous 48 states plus Washington, DC.

**Methods:**

Hierarchical logistic regression of resistance outcomes among *S. aureus* specimens to determine how much variation in the likelihood of resistance to five commonly used classes of antibiotics existed after accounting for patient-level characteristics.

**Results:**

The likelihood of drug resistance significantly varies across the VHA’s outpatient facilities, over and above the patient case mixture seen at each facility. In particular, VHA facilities in the US South and West regions have high likelihood of antibiotic resistance after accounting for patient factors.

**Conclusions:**

Suggest that community-level population or environmental characteristics are thus also associated with the likelihood of antimicrobial resistance in *S. aureus*. Integration of statistical and spatial analysis of antibiotic susceptibility testing results can help identify places with higher risk of drug-resistance, and thus populations facing limited treatment options, to ensure antibiotic stewardship or other policies have the greatest positive impact.

## Introduction

Rising resistance to non-beta lactam antimicrobial agents in *Staphylococcus aureus* can limit effective treatment options, particularly in empiric therapy prescribing. One tool used to better inform empiric therapy decisions by clinicians is cumulative susceptibility testing results, also known as antibiograms.^1–3^ Antibiograms summarize observed rates of susceptibility to different antimicrobial classes in *S. aureus* or other bacterial isolates in healthcare facilities over defined periods (usually annually), typically with isolates not disaggregated by infection site or inpatient versus outpatient settings.^4–10^ While the utility of antibiograms to accurately predict isolate susceptibility is questionable,^11,12^ they can be useful for another purpose: surveillance of antibiotic resistance rates in healthcare facilities at spatial resolutions more fine-scale than states.

While variance in facility-level, annual antibiograms has been observed, less understood is whether there exists variation in antibiotic resistance rates that is driven by factors other than the characteristics of patients treated at those facilities, that is the patient case mix. The likelihood of antibiotic-resistant *S. aureus* has been linked to patient-level attributes such as age, gender, prior antibiotic use, healthcare exposure, and comorbidities.^13–16^ There are also patient attributes, not well-captured in electronic health records, that can contribute to likelihood of infection and drug-resistant infection, such as socioeconomic status, occupational exposure to livestock, or crowded living conditions.^17–19^ It is possible that the variance in the prevalence of antimicrobial resistance (ie, antibiogram) at facility-level is primarily driven by these patient-level factors.

On the other hand, seasonality of infections, and potentially climatic variation in acquisition and maintenance of antibiotic resistance traits, suggests that there may also be non-random spatial variation in resistance rates, over and above the influence of individual patient attributes.^20–25^ Geographic variation in ecological-level sociodemographic factors such as wealth, healthcare accessibility, population density, and dominant occupation may also influence infection and antibiotic resistance likelihood. If significant variation exists, over and above the types of patients treated, and is not randomly distributed across the country, this suggests that antimicrobial surveillance programs need to examine underlying social or environmental drivers of resistance to understand where and why antimicrobial resistance is more or less likely, and our efforts to curve the rise of antimicrobial resistance should also focus on wider societal factors in addition to directly health-related issues of patients.

Utilizing data from the only nationwide provider of healthcare in the United States, the Veterans Health Administration (VHA), we sought to determine how much variation in the likelihood of resistance to five commonly used classes of antibiotics existed in outpatient *S. aureus* isolates after accounting for patient-level characteristics by hierarchical regression models. These quasi-risk-adjusted antibiograms can provide a clearer understanding of how drug resistance varies across the VHA’s outpatient facilities once patient case mix is controlled for.

### Data and methods

The VHA is the largest provider of integrated healthcare in the United States, serving over nine million veterans. Veterans are seen in outpatient settings at 1,181 Community Based Outpatient Clinics and 167 VA Health Care System facilities in the conterminous US. The VHA uses an integrated electronic health record and a nationwide data repository, the Corporate Data Warehouse (CDW). Approximately 90% of all facilities had on-site microbiology laboratories, and they are required to conduct quality control/quality assessment routinely per requirements of VHA-designated accreditation organizations and to use methods and equipment approved by the Food and Drug Administration.

Microbiology testing results for all *S. aureus* specimens isolated in outpatient VHA settings from 1/1/2021–12/31/2022 were accessed in the VHA CDW. Specimen date and facility location and source were recorded, as was resistance to the following classes: cephalosporins, clindamycin, macrolides, tetracyclines, trimethoprim-sulfamethoxazole (TMP-SMX). Microbiology data was linked to patient characteristics via the electronic health record. The first record per patient per year was retained for analysis. Patient age was calculated at time of specimen collection and categorized; self-report patient gender was also categorized. Prior antibiotic exposure in the 7 days, 8–30 days and 31–90 days before specimen collection was determined (1/0) separately for each antibiotic class (Table 1). Elixhauser comorbidity categories in two years prior to specimen date of collection were calculated.^26^

**Table 1.** Antibiotic classes used in calculation of prior antibiotic exposure

Model to predict resistance	Antibiotic exposure
Cephalosporins	All cephalosporins, penicillins, and carbapenems
Clindamycin	Clindamycin and macrolides
Macrolides	Clindamycin and macrolides
Tetracyclines	Tetracyclines
Trimethoprim-Sulfamethoxazole (TMP-SMX)	TMP/SMX

Models were generated annually (2021, 2022) for each of the five antibiotic classes; outcomes were resistance to each antibiotic class. Individual-level covariates for age, gender and prior antibiotic exposure were included. An indicator for month of year (1–12) was included to control for seasonal variation in *S. aureus* in VHA populations.^27^ Backward selection of Elixhauser categories was conducted in R using MASS and *lme4* ^28,29^ and comorbidities that were significant in both the 2021 and 2022 models for each antibiotic class were retained. The utility of a random intercept for facility to predict resistance was determined based upon comparison of model metrics (intraclass correlation coefficient (ICC), Akaike information criterion (AIC)) for full models with and without a random intercept and a null random intercept model for each of the five classes.

Final models were fit with random intercepts for facility, and odds ratios of resistance controlling for patient-level factors were generated for each facility.^30^ Models stratified by methicillin-resistant *Staphylococcus aureus* (MRSA)/methicillin-susceptible *Staphylococcus aureus* (MSSA), as defined by resistance to beta-lactam agents with antistaphylococcal activity, including intermediate resistance, to any cephalosporin (except for ceftaroline), antistaphylococcal penicillin, or carbapenem, were generated to determine differences in likelihood of resistance to non-beta lactams. Facility-level odds ratios from all models were charted to visualize differences in likelihood of resistance across VHA facilities after accounting for individual-level characteristics.

Mapping of odds ratios from overall models were used to visualize spatial heterogeneity in likelihoods of resistance across the US. To determine whether significant odds of resistance or susceptibility at facility-level, adjusted for individual patient covariates, were non-randomly spatially distributed, Moran’s I tests using eight nearest neighbors were generated. The Moran’s I index indicates whether high or low values, in this case adjusted odds ratios, are more spatially clustered than would be expected due to random chance.^31^ Spatial weighting based upon a set number of nearest neighbors is used because of the uneven spatial distribution of VHA facilities; neighbors defined using distance could result in some VHA facilities having no neighbors and others having a large number. Significance is defined at the alpha .05 level.

All statistical analysis was completed in R version 4.4.1 (R Foundation for Statistical Computing, Vienna, Austria) with glmmTMB package,^32^ and geographical analysis was completed in ArcGIS Pro 3.3.1 (Esri, Redlands, CA, USA). Ethical approval was given by the institutional review board at the University of Iowa and the Research and Development Committee at the Iowa City Veterans Affairs Health Care System with a waiver of informed consent because the study was a retrospective analysis of health records with no direct contact with patients. This study followed the Strengthening the Reporting of Observational Studies in Epidemiology reporting guideline.

## Results

A total of 46,686 specimens from 24,207 patients in 2021 and 22,659 patients in 2022 were included in the analysis (Table 2). Specimens were predominantly from male patients (*n* = 22,663, 94.3% in 2021, *n* = 21,381, 94.3% in 2022) and patients older than 65, and specimen sources were consistent with the outpatient nature of the data set. Rates of resistance varied by class, from a low of 4.4% of isolates resistant to TMP-SMX in 2021 to 38% resistant to macrolides in the same year. Rates of resistance by class were similar across the two years of data, except for macrolides resistance which dropped by 5%. Individuals with *S. aureus* isolates in outpatient settings had low rates of antibiotic exposure in the prior seven days, 30 days and 90 days; the highest rate of exposure was to cephalosporins in the prior 31–90 days in 2022 (*n* = 1,541, 6.8%). The prevalence of Elixhauser comorbidities, particularly hypertension (>75%) and diabetes (>50%), was high but stable among veteran outpatients (Supplemental Table 1).

**Table 2.** Characteristics of patients and *S. aureus* isolates included in the study. *Note*: The wound/other category for specimen source includes cerebrospinal fluid, feces, lower respiratory, and other

Variable	2021	2022
Specimens/Patients	24,027	22,659
Gender		
M	22,663 (94.3%)	21 381 (94.3%)
F	1,364 (5.7%)	1,278 (5.7%)
Age		
<45	2,019 (8.4%)	1,769 (7.8%)
45–54.99	2,224 (9.3%)	1,947 (8.6%)
55–64.99	4,893 (20.4%)	4,362 (19.2%)
65–74.99	8,672 (36.1%)	7,774 (34.3%)
75–84.99	4,402 (18.3%)	5,157 (22.8%)
>= 85	1,817 (7.5%)	1,650 (7.3%)
Source		
Blood	1,677 (7.0%)	1,485 (6.6%)
Nares/Pharynx	396 (1.6%)	430 (1.9%)
Synovial Fluid	233 (1.0%)	220 (1.0%)
Unknown	1,483 (6.2%)	1,312 (5.8%)
Urine	4,077 (17.0%)	3,979 (17.6%)
Wound/Other	16,161 (67.3%)	15,233 (67.2%)
Resistance		
Cephalosporins	8,915 (37.1%)	8,362 (34.8%)
Clindamycin	4,906 (20.4%)	4,664 (19.4%)
Macrolides	9,119 (38%)	7,976 (33.2%)
Tetracyclines	2,127 (8.9%)	2,178 (9.1%)
Trimethoprim-sulfamethoxazole (TMP-SMX)	1,050 (4.4%)	1,079 (4.5%)
Antibiotic exposure 7 days		
Cephalosporins	853 (3.6%)	964 (4.3%)
Clindamycin	277 (1.2%)	215 (.9%)
Macrolides	735 (3.1%)	772 (3.4%)
Tetracyclines	10 (.0%)	8 (.0%)
Trimethoprim-Sulfamethoxazole (TMP-SMX)	421 (1.8%)	408 (1.8%)
Antibiotic exposure 8–30 days		
Cephalosporins	662 (2.8%)	648 (2.9%)
Clindamycin	150 (.6%)	157 (.7%)
Macrolides	820 (3.4%)	887 (3.9%)
Tetracyclines	35 (.1%)	44 (.2%)
Trimethoprim-Sulfamethoxazole (TMP-SMX)	452 (1.9%)	412 (1.8%)
Antibiotic exposure 31–90 days		
Cephalosporins	1,425 (5.9%)	1,541 (6.8%)
Clindamycin	322 (1.3%)	283 (1.2%)
Macrolides	1,643 (6.8%)	1,653 (7.3%)
Tetracyclines	54 (.2%)	71 (.3%)
Trimethoprim-sulfamethoxazole (TMP-SMX)	811 (3.4%)	783 (3.5%)

ICCs for null and full random intercept models exhibited little variation across the five antibiotic classes in the overall analysis (Supplemental Table 2). This is indicative of a high degree of variability in outcomes within facilities. The AIC for full, random intercept models were lowest in both 2021 and 2022 across all five classes, signaling a small but important gain in ability to predict drug resistance when facility was considered. Results for overall and MRSA/MSSA stratified models are presented in Supplemental tables.^2–9^ Across the models, there is variation in the significance of patient-level characteristics such as age, gender and comorbidities. However, consistently across antibiotic classes and stratification and years and durations of exposure, prior antibiotic exposure is almost always associated with higher odds of a resistant *S. aureus* isolate.

**Figure 1.**
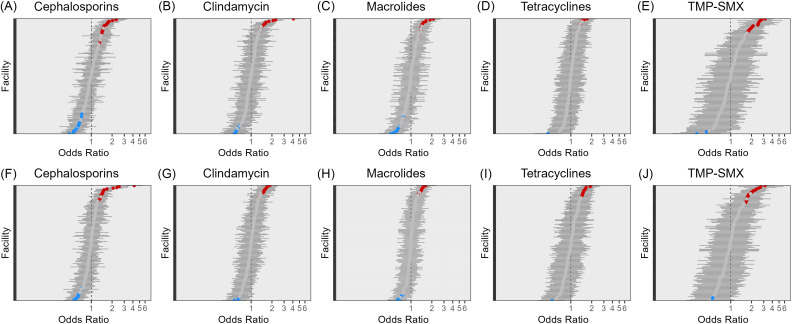
Adjusted odds ratios for antibiotic resistance in 2021 (A–E) and 2022 (F–J) for Veterans Health Administration (VHA) facilities across five antibiotic classes. Odds ratios that are significantly negative are shaded in blue, those that are significantly positive are shaded in red, insignificant odds ratios are gray.

After controlling for patient-level characteristics of gender, age, comorbidities, prior antibiotic exposure and season of isolate sample, facilities exhibited high degrees of variation in odds of antibiotic resistance across all five antibiotic classes (Figure 1). Most had insignificant odds in adjusted models, and positive odds ratios were more common than negative. The magnitude of significant, positive odds of resistance varied by antibiotic class: higher odds ratios of resistance were observed when examining TMP-SMX resistance than for classes such as tetracyclines.

**Figure 2.**
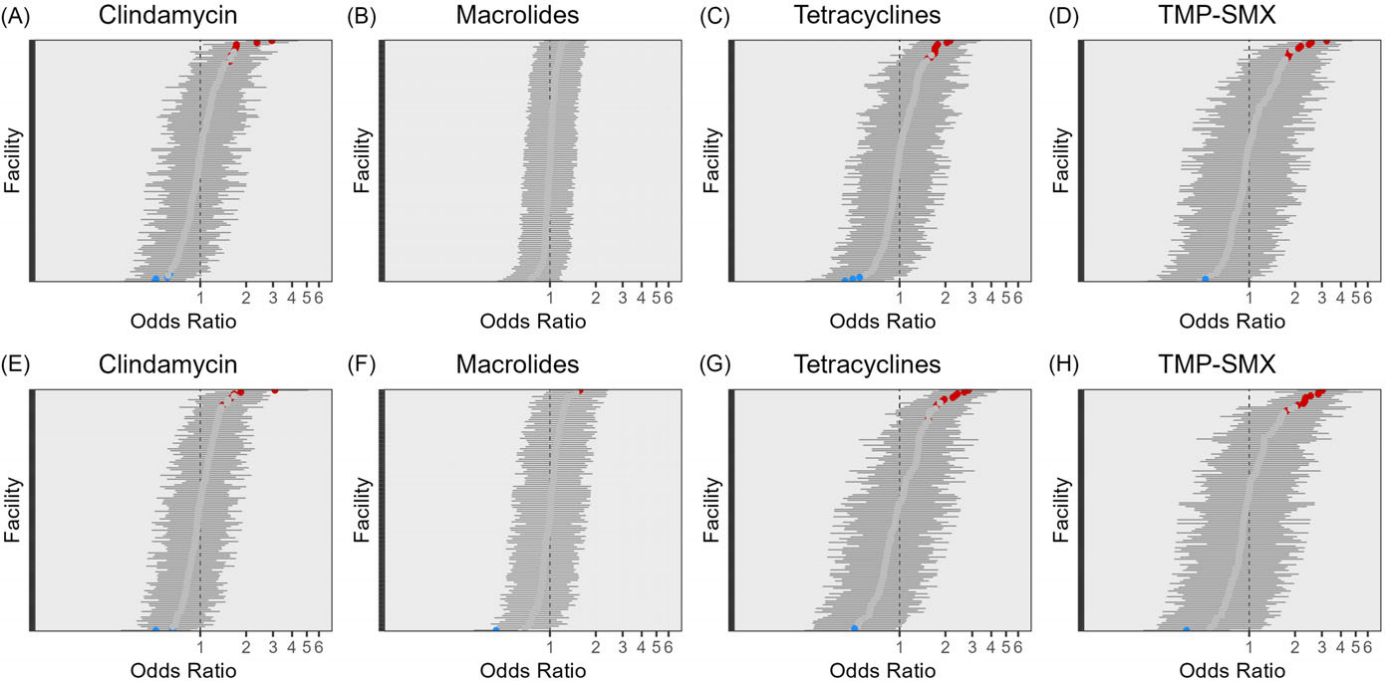
Adjusted odds ratios for antibiotic resistance in 2021 (A–D) and 2022 (E–H) in MRSA isolates for VHA facilities across five antibiotic classes. Odds ratios that are significantly negative are shaded in blue, those that are significantly positive are shaded in red, insignificant odds ratios are gray.

**Figure 3.**
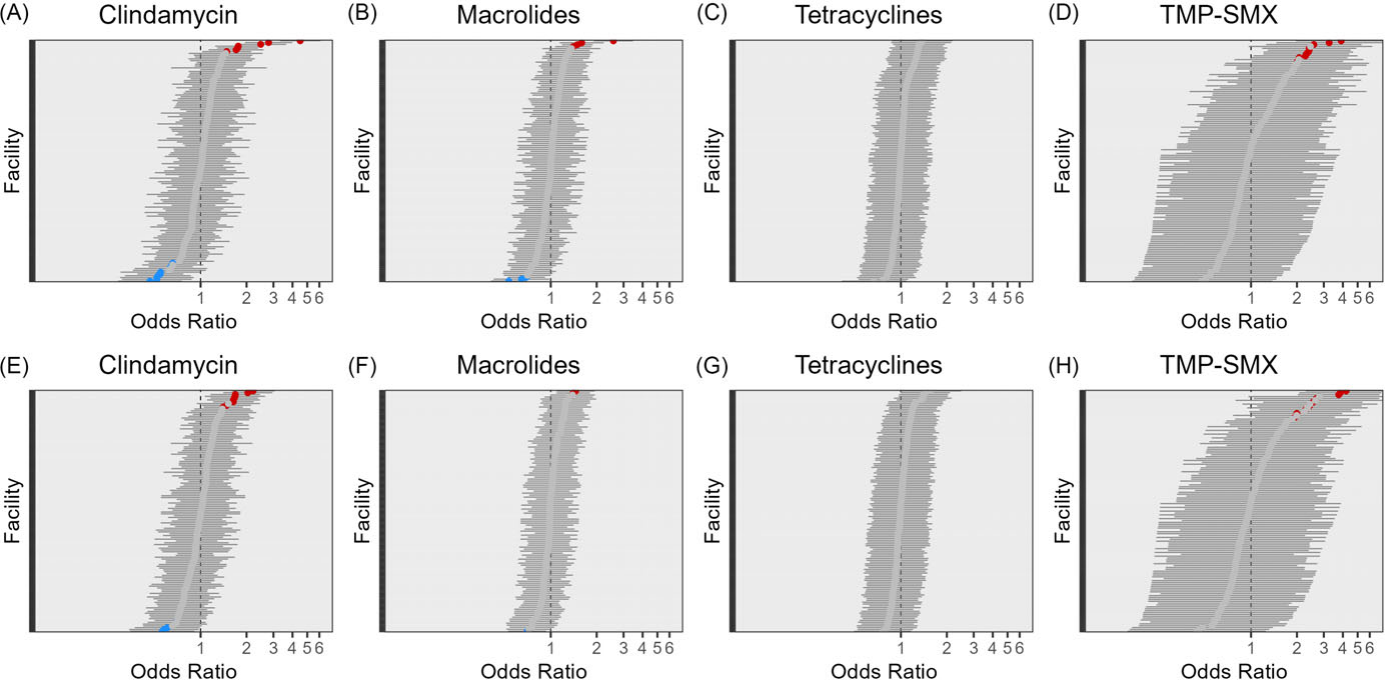
Adjusted odds ratios for antibiotic resistance in 2021 (A–D) and 2022 (E–H) in MSSA isolates for VHA facilities across five antibiotic classes. Odds ratios that are significantly negative are shaded in blue, those that are significantly positive are shaded in red, insignificant odds ratios are gray.

When stratified by MRSA/MSSA, differing odds for resistance to other antimicrobial classes were observed amongst VHA facilities in 2021 and 2022. No facilities had significantly high or low odds of macrolides resistance amongst MRSA isolates in 2021, and only one significantly high and one significantly low facility was detected in 2022 (Figure 2). Amongst MSSA isolates, no significantly high or low odds of tetracycline resistance were detected in VHA facilities once patient-level factors were accounted for (Figure 3). Additionally, no facility had lower odds of TMP-SMX resistance amongst MSSA isolates once patient-level factors were considered.

**Figure 4.**
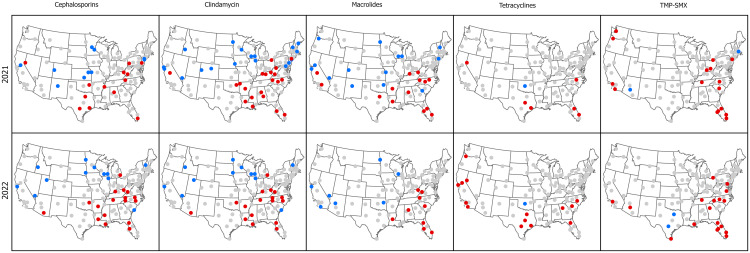
Adjusted odds ratios for antibiotic resistance in 2021 and 2022 for VHA facilities across five antibiotic classes. Odds ratios that are significantly negative are shaded in blue, those that are significantly positive are shaded in red, insignificant odds ratios are gray.

When adjusted odds ratios from the overall models were mapped, the general pattern was for positive odds of antibiotic resistance to occur in facilities in the South or Mid-Atlantic or far West, while negative odds of antibiotic resistance were found in northern facilities (Figure 4). Moran’s I tests indicated positive and statistically significant spatial clustering in both years for facility-level odds ratios for cephalosporins, macrolides and TMP-SMX; significant clustering of high and low facility-level odds ratios was also found in clindamycin odds ratios in 2021 and tetracycline odds ratios in 2022.

## Discussion

Among US veterans seen in outpatient settings with positive cultures for *S. aureus* in 2021 and 2022, rates of resistance to non-beta lactam antibiotics remained stable. This contrasts with trends observed in veteran populations from 2010–2019 that saw increasing tetracycline and TMP-SMX resistance.^27^ Of particular note, prior antibiotic exposure before the *S. aureus* date of detection was associated with significantly higher odds of resistance across all five classes analyzed, in alignment with other work on antimicrobial resistance risk in Gram-negative infections following prior antibiotic exposure.^33^ Modeling results indicated that likelihood of isolates being resistant to antimicrobial classes was associated with more than individual-level patient characteristics of age, gender, prior antibiotic exposure or comorbidities, or the month of the year when the sample was taken. Some VHA facilities had negative odds of drug-resistant *S. aureus* once accounting for patient-mix, while positive odds of drug resistance were more typically observed. Positive odds of resistance to tetracyclines were also more frequently observed amongst MRSA isolates in VHA facilities, consistent with prior analysis showing co-occurrence of resistance.^27^ Taken together, this suggests that community-level population or environment characteristics are associated with the likelihood of antimicrobial resistance in *S. aureus*.

When adjusted, significant positive or negative odds of resistance were mapped, facilities in the US South, Mid-Atlantic and West generally exhibited positive odds of resistance while lower adjusted odds were observed in Northern facilities. Analysis of the likelihood of neighboring facilities having similarly high or low odds indicated non-random distributions in VHA facilities for all but two year/class combinations. Prior work has indicated rising rates of resistance, particularly in tetracyclines and TMP-SMX, among veterans residing in Southern and Western counties.^27^ High odds of resistant isolates, over and above patient case mix, could be driven by sociodemographic factors such as poverty and crowding, or climatic factors not accounted for by month-of-year in the models, such as heat and humidity, or combinations thereof.^17,18,24,34–36^ Our findings highlight the need to consider the social and environmental context of patients when understanding drivers of antimicrobial resistance.

Study limitations are those inherent in the use of VHA data and EHR data. While the VHA is the largest, integrated provider of care in the US, with standardized microbiology testing linked to health histories, the typical patient profile of the VHA is not representative of the larger US population. VHA patients tend to be more male gender, older age and White race. Within VHA records it is also difficult to adequately characterize wealth. EHR can capture a high degree of information on prior antibiotic use and comorbidities but cannot assess how household, occupational, recreational or other community exposures interact to increase or decrease likelihood of *S. aureus*, or drug resistance in isolates. Finally, VHA data aggregated to facility means that veterans from multiple cities, counties and across the rural-urban divided are assigned to a singular point location. Future work should be conducted at other levels of patient aggregation to better understand those population and environment factors, other than patient-level characteristics, that may be responsible for the structured variation in antimicrobial risk observed in this research.

Enhancing the utility of summarized antimicrobial susceptibility/resistance results to inform surveillance in the US is critical to mitigating future increases in antibiotic resistance. Methods that account for both the influence of individuals as well as the communities from which they are drawn will improve the accuracy of our estimation of rising, falling or stable rates of resistance as well as, potentially, identify levers outside of provider prescribing that can be utilized to decrease resistance rates. The integration of statistical and spatial analysis of antibiotic susceptibility testing results is vital to identifying peoples and places where policies or programs to remediate inequities in resistant bacteria, and thus limited treatment options, would have the greatest impact.

## Supporting information

10.1017/ash.2025.10127.sm001 Carrel et al. supplementary material Carrel et al. supplementary material

## Data Availability

The datasets generated in this study are not publicly available due to their sensitive nature and ownership by the VHA.

## Supplementary material

The supplementary material for this article can be found at https://doi.org/10.1017/ash.2025.10127.
