# Supplementary material for: Variation in facility-level likelihood of drug-resistant Staphylococcus aureus in outpatients remains after patient-level risk adjustment
Source: Antimicrob Steward Healthc Epidemiol. 2025 Sep 10;5(1):e205. doi: 10.1017/ash.2025.10127 (PMC12451804; doi:10.1017/ash.2025.10127)
Supplement: Carrel et al. supplementary material [file S2732494X25101277sup001.docx]

**Supplemental Tables**

Supplemental Table 1. Elixhauser comborbidities by year among patients with positive *S. aureus* cultures.

| **Variable** | **2021** | **2022** |
| --- | --- | --- |
| Specimens/Patients. | 24027 | 22659 |
| Comorbidities |  |  |
| Alcohol Abuse | 3605 (15%) | 3358 (14.8%) |
| Anemia Deficiency | 4003 (16.7%) | 4014 (17.7%) |
| Arrhythmia | 7773 (32.4%) | 7583 (33.5%) |
| Blood Loss Anemia | 712 (3%) | 687 (3%) |
| Congestive Heart Failure | 5262 (21.9%) | 5037 (22.2%) |
| Chronic Pulmonary | 7194 (29.9%) | 6790 (30%) |
| Coagulopathy | 1710 (7.1%) | 1625 (7.2%) |
| Depression | 9438 (39.3%) | 9063 (40%) |
| Diabetes mellitus | 12611 (52.5%) | 12144 (53.6%) |
| Drug Abuse | 3087 (12.8%) | 2926 (12.9%) |
| Fluid Electrolyte | 6962 (29%) | 6761 (29.8%) |
| HIV/AIDS | 322 (1.3%) | 297 (1.3%) |
| Hypertension | 18172 (75.6%) | 17312 (76.4%) |
| Hypothyroidism | 3008 (12.5%) | 2922 (12.9%) |
| Liver Disease | 2918 (12.1%) | 2925 (12.9%) |
| Lymphoma | 403 (1.7%) | 433 (1.9%) |
| Metastatic Cancer | 815 (3.4%) | 801 (3.5%) |
| Neurological | 3702 (15.4%) | 3591 (15.8%) |
| Non-Metastatic Cancer | 3658 (15.2%) | 3618 (16%) |
| Obesity | 7512 (31.3%) | 7049 (31.1%) |
| Paralysis | 1174 (4.9%) | 1095 (4.8%) |
| Peptic Ulcer | 381 (1.6%) | 354 (1.6%) |
| Psychoses | 993 (4.1%) | 925 (4.1%) |
| Pulmonary Circulation Disorder | 1592 (6.6%) | 1591 (7%) |
| Peripheral Vascular Disease | 6223 (25.9%) | 6085 (26.9%) |
| Renal Failure | 5982 (24.9%) | 5812 (25.6%) |
| Rheumatoid Arthritis | 1116 (4.6%) | 1184 (5.2%) |
| Valvular Disease | 2068 (8.6%) | 2073 (9.1%) |
| Weight Loss | 2618 (10.9%) | 2597 (11.5%) |

Supplemental Table 2. Intraclass correlation coefficients (ICCs) and Akaike information criterion (AICs) for full models with and without a random intercept and a null, random intercept model. TMP-SMX: Trimethoprim-Sulfamethoxazole; RI: Random Intercept

|  | **2021** | | | | | **2022** | | | | |
| --- | --- | --- | --- | --- | --- | --- | --- | --- | --- | --- |
| **ICC** | Cephalosporins | Clindamycin | Macrolides | Tetracyclines | TMP-SMX | Cephalosporins | Clindamycin | Macrolides | Tetracyclines | TMP-SMX |
| Full-No RI | ----- | ----- | ----- | ----- | ----- | ----- | ----- | ----- | ----- | ----- |
| Null-RI | 0.035 | 0.033 | 0.028 | 0.023 | 0.085 | 0.031 | 0.026 | 0.018 | 0.035 | 0.077 |
| Full-RI | 0.036 | 0.034 | 0.028 | 0.023 | 0.088 | 0.032 | 0.025 | 0.017 | 0.035 | 0.076 |
| **AIC** |  |  |  |  |  |  |  |  |  |  |
| Full-No RI | 30882.0 | 21427.9 | 22767.56 | 13943.7 | 8402.6 | 29066.1 | 20265.4 | 20492.06 | 13932.6 | 8466.3 |
| Null-RI | 31161.5 | 21740.7 | 22905.02 | 14083.3 | 8353.9 | 29332.0 | 20631.8 | 20764.12 | 14010.9 | 8416.8 |
| Full-RI | 30531.2 | 21158.4 | 22578.22 | 13898.8 | 8240.4 | 28753.4 | 20143.7 | 20410.99 | 13834.4 | 8324.5 |

Supplemental Table 2. Overall model results for cephalosporin resistance.

|  | Cephalosporin 2021 | | | Cephalosporin 2022 | | |
| --- | --- | --- | --- | --- | --- | --- |
| *Predictors* | Null | Full RI | Full no RI | Null | Full RI | Full no RI |
| (Intercept) | 0.58 (0.54 – 0.62) | 0.4 (0.34 – 0.48) | 0.42 (0.35 – 0.49) | 0.58 (0.54 – 0.61) | 0.42 (0.35 – 0.50) | 0.42 (0.35 – 0.49) |
| monthofyear [2] |  | 1.09 (0.95 – 1.25) | 1.09 (0.96 – 1.25) |  | 0.93 (0.81 – 1.06) | 0.95 (0.83 – 1.08) |
| monthofyear [3] |  | 0.94 (0.83 – 1.07) | 0.94 (0.83 – 1.06) |  | 0.88 (0.78 – 1.00) | 0.9 (0.79 – 1.02) |
| monthofyear [4] |  | 1.1 (0.97 – 1.25) | 1.09 (0.96 – 1.24) |  | 0.9 (0.79 – 1.03) | 0.93 (0.82 – 1.06) |
| monthofyear [5] |  | 1.09 (0.96 – 1.24) | 1.08 (0.95 – 1.23) |  | 0.87 (0.76 – 0.99) | 0.9 (0.79 – 1.02) |
| monthofyear [6] |  | 1 (0.88 – 1.14) | 1 (0.88 – 1.13) |  | 0.9 (0.78 – 1.02) | 0.94 (0.82 – 1.07) |
| monthofyear [7] |  | 1.04 (0.91 – 1.18) | 1.03 (0.90 – 1.17) |  | 0.95 (0.83 – 1.08) | 0.99 (0.87 – 1.13) |
| monthofyear [8] |  | 1.03 (0.90 – 1.17) | 1.03 (0.90 – 1.17) |  | 0.82 (0.71 – 0.93) | 0.85 (0.75 – 0.97) |
| monthofyear [9] |  | 1.03 (0.90 – 1.18) | 1.02 (0.89 – 1.16) |  | 0.93 (0.82 – 1.07) | 0.96 (0.84 – 1.10) |
| monthofyear [10] |  | 1.03 (0.91 – 1.18) | 1.03 (0.91 – 1.18) |  | 0.92 (0.80 – 1.06) | 0.95 (0.83 – 1.09) |
| monthofyear [11] |  | 0.98 (0.86 – 1.12) | 0.98 (0.86 – 1.12) |  | 0.95 (0.83 – 1.09) | 0.99 (0.87 – 1.13) |
| monthofyear [12] |  | 1.07 (0.93 – 1.22) | 1.06 (0.93 – 1.21) |  | 0.95 (0.82 – 1.08) | 0.97 (0.85 – 1.11) |
| Gender [M] |  | 1.16 (1.02 – 1.31) | 1.15 (1.01 – 1.29) |  | 1.13 (0.99 – 1.28) | 1.11 (0.98 – 1.26) |
| age_cat45-55 |  | 0.94 (0.83 – 1.08) | 0.96 (0.84 – 1.09) |  | 0.95 (0.82 – 1.09) | 0.96 (0.84 – 1.11) |
| age_cat55-65 |  | 0.95 (0.84 – 1.07) | 0.93 (0.82 – 1.04) |  | 1 (0.88 – 1.13) | 1 (0.88 – 1.13) |
| age_cat65-75 |  | 0.94 (0.84 – 1.06) | 0.9 (0.80 – 1.01) |  | 1.07 (0.94 – 1.21) | 1.05 (0.93 – 1.19) |
| age_cat75-85 |  | 1.1 (0.97 – 1.25) | 1.03 (0.91 – 1.17) |  | 1.16 (1.02 – 1.32) | 1.14 (1.00 – 1.30) |
| age cat [GT85] |  | 1.31 (1.13 – 1.51) | 1.21 (1.05 – 1.40) |  | 1.35 (1.15 – 1.58) | 1.3 (1.11 – 1.51) |
| Elix parlysis |  | 1.63 (1.44 – 1.85) | 1.57 (1.39 – 1.77) |  | 1.62 (1.42 – 1.84) | 1.6 (1.41 – 1.82) |
| Elix neurological |  | 1.22 (1.13 – 1.32) | 1.22 (1.13 – 1.31) |  | 1.23 (1.14 – 1.33) | 1.21 (1.12 – 1.31) |
| Elix chronicpulmonary |  | 1.15 (1.08 – 1.22) | 1.18 (1.11 – 1.26) |  | 1.11 (1.05 – 1.19) | 1.13 (1.06 – 1.20) |
| Elix HIVAIDS |  | 1.68 (1.33 – 2.11) | 1.61 (1.29 – 2.02) |  | 2.04 (1.60 – 2.60) | 2.01 (1.59 – 2.55) |
| Elix lymphoma |  | 0.86 (0.70 – 1.07) | 0.85 (0.69 – 1.05) |  | 0.83 (0.67 – 1.02) | 0.83 (0.67 – 1.02) |
| Elix obesity |  | 0.92 (0.86 – 0.97) | 0.91 (0.86 – 0.97) |  | 0.97 (0.91 – 1.03) | 0.95 (0.89 – 1.01) |
| Elix deficiencyanemia |  | 1.08 (1.01 – 1.17) | 1.09 (1.01 – 1.18) |  | 1.11 (1.03 – 1.20) | 1.12 (1.04 – 1.21) |
| Elix psychoses |  | 1.26 (1.10 – 1.45) | 1.25 (1.09 – 1.43) |  | 1.21 (1.05 – 1.39) | 1.21 (1.05 – 1.38) |
| Elix depression |  | 1.14 (1.07 – 1.20) | 1.14 (1.07 – 1.21) |  | 1.13 (1.06 – 1.20) | 1.12 (1.06 – 1.19) |
| Elix drugabuse |  | 1.34 (1.23 – 1.46) | 1.33 (1.22 – 1.45) |  | 1.41 (1.29 – 1.54) | 1.39 (1.28 – 1.52) |
| ceph ant pen 1 4 D7 |  | 1.22 (1.05 – 1.42) | 1.22 (1.05 – 1.41) |  | 1.2 (1.04 – 1.38) | 1.22 (1.06 – 1.40) |
| ceph ant pen 1 4 D30 |  | 1.34 (1.13 – 1.60) | 1.36 (1.14 – 1.61) |  | 1.22 (1.02 – 1.46) | 1.21 (1.02 – 1.45) |
| ceph ant pen 1 4 D90 |  | 1.57 (1.39 – 1.77) | 1.56 (1.38 – 1.75) |  | 1.47 (1.31 – 1.65) | 1.47 (1.31 – 1.65) |
| Penicillins D7 |  | 0.9 (0.50 – 1.62) | 0.87 (0.48 – 1.54) |  | 0.76 (0.41 – 1.42) | 0.75 (0.40 – 1.37) |
| Penicillins D30 |  | 1.18 (0.82 – 1.70) | 1.14 (0.79 – 1.63) |  | 1.5 (1.05 – 2.16) | 1.48 (1.04 – 2.11) |
| Penicillins D90 |  | 1 (0.79 – 1.27) | 0.99 (0.78 – 1.24) |  | 1.26 (1.00 – 1.60) | 1.24 (0.98 – 1.56) |
| Carbapenems D7 |  | 0.89 (0.43 – 1.82) | 0.93 (0.45 – 1.88) |  | 1.59 (0.80 – 3.15) | 1.51 (0.77 – 3.02) |
| Carbapenems D30 |  | 0.95 (0.46 – 1.96) | 1 (0.49 – 2.08) |  | 2.14 (1.02 – 4.47) | 2.23 (1.11 – 4.78) |
| Carbapenems D90 |  | 1.97 (1.37 – 2.82) | 1.98 (1.40 – 2.84) |  | 1.83 (1.27 – 2.64) | 1.87 (1.31 – 2.70) |
| Elix metastatic |  | 0.85 (0.73 – 0.99) | 0.85 (0.73 – 0.99) |  | 0.81 (0.69 – 0.94) | 0.82 (0.70 – 0.95) |
| Elix rheumatoid |  | 0.83 (0.72 – 0.94) | 0.83 (0.73 – 0.94) |  | 0.87 (0.76 – 0.98) | 0.87 (0.76 – 0.98) |
| Elix weightloss |  | 1.13 (1.03 – 1.24) | 1.12 (1.02 – 1.23) |  | 1.2 (1.10 – 1.32) | 1.19 (1.09 – 1.30) |
| Elix fluidelectrolyte |  | 1.24 (1.16 – 1.32) | 1.22 (1.15 – 1.31) |  | 1.15 (1.07 – 1.23) | 1.15 (1.07 – 1.22) |
| Elix htn any |  | 0.89 (0.83 – 0.96) | 0.92 (0.86 – 0.99) |  | 0.91 (0.84 – 0.98) | 0.94 (0.87 – 1.01) |
| *Random Effects* |  |  |  |  |  |  |
| σ2 | 3.29 | 3.29 |  | 3.29 | 3.29 |  |
| τ00 | 0.119 | 0.124 |  | 0.106 | 0.107 |  |
| ICC | 0.035 | 0.036 |  | 0.031 | 0.032 |  |
| Marginal R2 / Conditional R2 | 0.000 / 0.035 | 0.038 / 0.073 | 0.03 | 0.000 / 0.031 | 0.038 / 0.068 | 0.03 |
| AIC | 31161.531 | 30531.222 | 30882.002 | 29331.989 | 28753.416 | 29066.137 |

Supplemental Table 3. Overall model results for clindamycin resistance.

|  | Clindamycin 2021 | | | | Clindamycin 2022 | | | |
| --- | --- | --- | --- | --- | --- | --- | --- | --- |
| *Predictors* | Null | Full RI | Full no RI | Null | | Full RI | Full no RI |  |
| (Intercept) | 0.32 (0.30 – 0.35) | 0.23 (0.19 – 0.29) | 0.25 (0.21 – 0.31) | 0.33 (0.31 – 0.35) | | 0.22 (0.17 – 0.27) | 0.22 (0.18 – 0.27) |  |
| monthofyear [2] |  | 0.94 (0.80 – 1.10) | 0.92 (0.79 – 1.08) |  | | 0.99 (0.84 – 1.17) | 1.01 (0.85 – 1.18) |  |
| monthofyear [3] |  | 1.02 (0.87 – 1.18) | 0.99 (0.86 – 1.16) |  | | 1.05 (0.90 – 1.23) | 1.06 (0.91 – 1.24) |  |
| monthofyear [4] |  | 0.91 (0.78 – 1.06) | 0.89 (0.77 – 1.04) |  | | 0.96 (0.82 – 1.13) | 0.96 (0.82 – 1.13) |  |
| monthofyear [5] |  | 1.01 (0.86 – 1.18) | 0.99 (0.85 – 1.16) |  | | 0.97 (0.82 – 1.14) | 0.98 (0.84 – 1.15) |  |
| monthofyear [6] |  | 0.86 (0.74 – 1.01) | 0.84 (0.72 – 0.97) |  | | 1.12 (0.96 – 1.32) | 1.14 (0.97 – 1.33) |  |
| monthofyear [7] |  | 0.81 (0.69 – 0.95) | 0.8 (0.68 – 0.93) |  | | 0.89 (0.75 – 1.05) | 0.91 (0.77 – 1.07) |  |
| monthofyear [8] |  | 0.86 (0.74 – 1.01) | 0.86 (0.73 – 1.00) |  | | 0.97 (0.82 – 1.14) | 0.99 (0.84 – 1.16) |  |
| monthofyear [9] |  | 0.83 (0.71 – 0.98) | 0.83 (0.71 – 0.97) |  | | 1 (0.85 – 1.18) | 1.01 (0.86 – 1.19) |  |
| monthofyear [10] |  | 0.79 (0.68 – 0.94) | 0.79 (0.67 – 0.92) |  | | 1.02 (0.86 – 1.20) | 1.02 (0.86 – 1.20) |  |
| monthofyear [11] |  | 0.81 (0.68 – 0.95) | 0.8 (0.68 – 0.94) |  | | 1.01 (0.86 – 1.20) | 1.03 (0.87 – 1.22) |  |
| monthofyear [12] |  | 0.79 (0.67 – 0.93) | 0.78 (0.67 – 0.92) |  | | 1.02 (0.86 – 1.21) | 1.03 (0.87 – 1.22) |  |
| Gender [M] |  | 0.96 (0.83 – 1.12) | 0.94 (0.81 – 1.09) |  | | 1 (0.85 – 1.16) | 1.01 (0.87 – 1.17) |  |
| age_cat45-55 |  | 1 (0.85 – 1.18) | 1.01 (0.86 – 1.18) |  | | 1.04 (0.88 – 1.23) | 1.04 (0.88 – 1.23) |  |
| age_cat55-65 |  | 0.99 (0.86 – 1.15) | 0.98 (0.85 – 1.13) |  | | 0.96 (0.83 – 1.12) | 0.96 (0.83 – 1.11) |  |
| age_cat65-75 |  | 1.09 (0.94 – 1.25) | 1.06 (0.92 – 1.22) |  | | 1 (0.87 – 1.16) | 0.98 (0.85 – 1.14) |  |
| age_cat75-85 |  | 1.25 (1.08 – 1.46) | 1.21 (1.04 – 1.40) |  | | 1.07 (0.91 – 1.25) | 1.04 (0.89 – 1.21) |  |
| age cat [GT85] |  | 1.43 (1.19 – 1.72) | 1.36 (1.14 – 1.63) |  | | 1.4 (1.15 – 1.69) | 1.34 (1.11 – 1.62) |  |
| Clindamycin D7 |  | 1.49 (1.12 – 1.97) | 1.44 (1.09 – 1.89) |  | | 1.88 (1.39 – 2.56) | 1.88 (1.39 – 2.55) |  |
| Clindamycin D30 |  | 7.18 (4.72 – 10.93) | 7.04 (4.70 – 10.79) |  | | 4.64 (3.16 – 6.82) | 4.57 (3.14 – 6.73) |  |
| Clindamycin D90 |  | 2.01 (1.55 – 2.59) | 2.06 (1.60 – 2.65) |  | | 1.68 (1.28 – 2.20) | 1.69 (1.29 – 2.20) |  |
| Macrolides D7 |  | 1.12 (0.94 – 1.34) | 1.13 (0.94 – 1.34) |  | | 1.14 (0.96 – 1.36) | 1.15 (0.97 – 1.36) |  |
| Macrolides D30 |  | 1.77 (1.49 – 2.09) | 1.72 (1.46 – 2.03) |  | | 1.82 (1.55 – 2.15) | 1.77 (1.50 – 2.08) |  |
| Macrolides D90 |  | 1.44 (1.27 – 1.63) | 1.42 (1.26 – 1.61) |  | | 1.48 (1.31 – 1.68) | 1.49 (1.31 – 1.68) |  |
| Elix pvd |  | 1.21 (1.12 – 1.31) | 1.22 (1.13 – 1.32) |  | | 1.14 (1.06 – 1.24) | 1.16 (1.07 – 1.26) |  |
| Elix parlysis |  | 1.55 (1.31 – 1.83) | 1.54 (1.30 – 1.81) |  | | 1.44 (1.21 – 1.71) | 1.4 (1.18 – 1.66) |  |
| Elix neurological |  | 1.16 (1.05 – 1.28) | 1.15 (1.05 – 1.27) |  | | 1.14 (1.03 – 1.25) | 1.13 (1.03 – 1.25) |  |
| Elix chronicpulmonary |  | 1.17 (1.08 – 1.26) | 1.17 (1.08 – 1.25) |  | | 1.11 (1.03 – 1.20) | 1.12 (1.04 – 1.20) |  |
| Elix DM any |  | 1.09 (1.01 – 1.18) | 1.11 (1.03 – 1.19) |  | | 1.15 (1.07 – 1.24) | 1.16 (1.07 – 1.25) |  |
| Elix renalfailure |  | 1.14 (1.05 – 1.24) | 1.14 (1.05 – 1.24) |  | | 1.15 (1.06 – 1.25) | 1.15 (1.06 – 1.25) |  |
| Elix pepticulcer |  | 0.74 (0.56 – 0.98) | 0.72 (0.54 – 0.95) |  | | 0.74 (0.56 – 0.99) | 0.76 (0.57 – 1.01) |  |
| Elix HIVAIDS |  | 1.55 (1.19 – 2.03) | 1.6 (1.23 – 2.07) |  | | 1.52 (1.16 – 1.99) | 1.55 (1.18 – 2.01) |  |
| Elix lymphoma |  | 1.22 (0.96 – 1.55) | 1.22 (0.96 – 1.54) |  | | 1.27 (1.00 – 1.62) | 1.27 (0.99 – 1.60) |  |
| Elix obesity |  | 0.96 (0.89 – 1.03) | 0.95 (0.88 – 1.02) |  | | 0.92 (0.85 – 0.99) | 0.91 (0.85 – 0.99) |  |
| Elix deficiencyanemia |  | 1.16 (1.06 – 1.27) | 1.15 (1.05 – 1.26) |  | | 1.17 (1.07 – 1.28) | 1.18 (1.08 – 1.29) |  |
| Elix psychoses |  | 1.2 (1.01 – 1.41) | 1.2 (1.02 – 1.41) |  | | 1.23 (1.04 – 1.44) | 1.23 (1.05 – 1.45) |  |
| Elix depression |  | 1.15 (1.07 – 1.23) | 1.15 (1.07 – 1.23) |  | | 1.16 (1.07 – 1.24) | 1.15 (1.07 – 1.24) |  |
| *Random Effects* |  |  |  |  | |  |  |  |
| σ2 | 3.29 | 3.29 |  | 3.29 | | 3.29 |  |  |
| τ00 | 0.112 | 0.114 StaPa |  | 0.086 | | 0.085 |  |  |
| ICC | 0.033 | 0.034 |  | 0.026 | | 0.025 |  |  |
| Marginal R2 / Conditional R2 | 0.000 / 0.033 | 0.049 / 0.081 | 0.037 | 0.000 / 0.026 | | 0.043 / 0.067 | 0.034 |  |
| AIC | 21740.68 | 21158.424 | 21427.882 | 20631.846 | | 20143.711 | 20265.419 |  |

Supplemental Table 4. Overall model results for macrolides resistance.

|  | Macrolides 2021 | | | Macrolides 2022 | | |
| --- | --- | --- | --- | --- | --- | --- |
| *Predictors* | Null | Full RI | Full no RI | Null | Full RI | Full no RI |
| (Intercept) | 1.15 (1.08 – 1.23) | 0.93 (0.76 – 1.14) | 0.95 (0.79 – 1.15) | 1.1 (1.04 – 1.17) | 0.91 (0.74 – 1.12) | 0.91 (0.75 – 1.11) |
| monthofyear [2] |  | 0.95 (0.82 – 1.11) | 0.97 (0.84 – 1.13) |  | 1.02 (0.87 – 1.20) | 1.04 (0.89 – 1.21) |
| monthofyear [3] |  | 1 (0.87 – 1.16) | 1.01 (0.87 – 1.16) |  | 1 (0.86 – 1.17) | 1.01 (0.87 – 1.18) |
| monthofyear [4] |  | 1.1 (0.95 – 1.27) | 1.11 (0.96 – 1.28) |  | 1.04 (0.89 – 1.21) | 1.05 (0.90 – 1.23) |
| monthofyear [5] |  | 1.02 (0.88 – 1.18) | 1.02 (0.88 – 1.18) |  | 0.96 (0.82 – 1.12) | 0.97 (0.83 – 1.12) |
| monthofyear [6] |  | 0.96 (0.83 – 1.11) | 0.96 (0.83 – 1.11) |  | 0.96 (0.82 – 1.12) | 0.98 (0.84 – 1.14) |
| monthofyear [7] |  | 1.01 (0.87 – 1.17) | 1.02 (0.88 – 1.18) |  | 0.93 (0.80 – 1.09) | 0.95 (0.81 – 1.10) |
| monthofyear [8] |  | 1 (0.86 – 1.17) | 1.02 (0.88 – 1.19) |  | 0.89 (0.76 – 1.04) | 0.9 (0.77 – 1.04) |
| monthofyear [9] |  | 1.05 (0.90 – 1.23) | 1.07 (0.92 – 1.24) |  | 0.96 (0.82 – 1.13) | 0.97 (0.83 – 1.14) |
| monthofyear [10] |  | 0.97 (0.84 – 1.13) | 1 (0.86 – 1.16) |  | 0.98 (0.84 – 1.15) | 0.99 (0.85 – 1.16) |
| monthofyear [11] |  | 0.87 (0.74 – 1.01) | 0.89 (0.76 – 1.04) |  | 1.01 (0.86 – 1.18) | 1.02 (0.87 – 1.20) |
| monthofyear [12] |  | 1.02 (0.87 – 1.19) | 1.03 (0.88 – 1.20) |  | 1.07 (0.91 – 1.25) | 1.08 (0.92 – 1.27) |
| Gender [M] |  | 0.96 (0.83 – 1.10) | 0.96 (0.83 – 1.10) |  | 0.96 (0.83 – 1.11) | 0.96 (0.83 – 1.11) |
| age_cat45-55 |  | 0.99 (0.85 – 1.14) | 1.02 (0.88 – 1.17) |  | 0.99 (0.85 – 1.16) | 1.01 (0.87 – 1.18) |
| age_cat55-65 |  | 1 (0.89 – 1.14) | 1.02 (0.90 – 1.15) |  | 0.95 (0.83 – 1.09) | 0.97 (0.84 – 1.10) |
| age_cat65-75 |  | 0.99 (0.87 – 1.11) | 0.98 (0.87 – 1.10) |  | 1 (0.88 – 1.14) | 1 (0.88 – 1.13) |
| age_cat75-85 |  | 1.09 (0.96 – 1.25) | 1.08 (0.95 – 1.24) |  | 1.06 (0.92 – 1.22) | 1.06 (0.93 – 1.22) |
| age cat [GT85] |  | 1.36 (1.15 – 1.61) | 1.34 (1.14 – 1.58) |  | 1.35 (1.12 – 1.61) | 1.34 (1.12 – 1.60) |
| Clindamycin D7 |  | 1.46 (1.09 – 1.96) | 1.49 (1.12 – 2.01) |  | 1.48 (1.05 – 2.08) | 1.49 (1.06 – 2.10) |
| Clindamycin D30 |  | 2.61 (1.67 – 4.09) | 2.6 (1.69 – 4.13) |  | 2.56 (1.61 – 4.06) | 2.5 (1.60 – 4.03) |
| Clindamycin D90 |  | 1.3 (0.99 – 1.70) | 1.28 (0.99 – 1.68) |  | 1.22 (0.91 – 1.65) | 1.22 (0.90 – 1.64) |
| Macrolides D7 |  | 1.18 (0.99 – 1.42) | 1.24 (1.03 – 1.48) |  | 1.2 (1.01 – 1.44) | 1.24 (1.04 – 1.48) |
| Macrolides D30 |  | 1.48 (1.22 – 1.78) | 1.46 (1.21 – 1.76) |  | 1.8 (1.49 – 2.18) | 1.76 (1.46 – 2.13) |
| Macrolides D90 |  | 1.58 (1.38 – 1.80) | 1.59 (1.39 – 1.81) |  | 1.55 (1.36 – 1.78) | 1.58 (1.38 – 1.81) |
| Elix parlysis |  | 1.45 (1.22 – 1.73) | 1.42 (1.20 – 1.69) |  | 1.37 (1.14 – 1.64) | 1.36 (1.14 – 1.63) |
| Elix neurological |  | 1.16 (1.05 – 1.27) | 1.16 (1.06 – 1.27) |  | 1.15 (1.05 – 1.27) | 1.15 (1.04 – 1.27) |
| Elix chronicpulmonary |  | 1.2 (1.12 – 1.29) | 1.2 (1.12 – 1.28) |  | 1.23 (1.14 – 1.33) | 1.25 (1.16 – 1.34) |
| Elix HIVAIDS |  | 1.98 (1.48 – 2.66) | 2.01 (1.51 – 2.69) |  | 2.59 (1.87 – 3.57) | 2.55 (1.86 – 3.54) |
| Elix obesity |  | 0.9 (0.84 – 0.97) | 0.9 (0.84 – 0.96) |  | 0.88 (0.82 – 0.95) | 0.88 (0.82 – 0.95) |
| Elix psychoses |  | 1.19 (1.01 – 1.40) | 1.17 (1.00 – 1.38) |  | 1.23 (1.04 – 1.45) | 1.21 (1.02 – 1.43) |
| Elix depression |  | 1.15 (1.07 – 1.23) | 1.15 (1.07 – 1.23) |  | 1.07 (1.00 – 1.15) | 1.08 (1.01 – 1.16) |
| Elix drugabuse |  | 1.34 (1.22 – 1.48) | 1.33 (1.21 – 1.47) |  | 1.39 (1.25 – 1.54) | 1.38 (1.24 – 1.52) |
| *Random Effects* |  |  |  |  |  |  |
| σ2 | 3.29 | 3.29 |  | 3.29 | 3.29 |  |
| τ00 | 0.093 | 0.095 |  | 0.059 | 0.056 |  |
| ICC | 0.028 | 0.028 |  | 0.018 | 0.017 |  |
| Marginal R2 / Conditional R2 | 0.000 / 0.028 | 0.031 / 0.058 | 0.023 | 0.000 / 0.018 | 0.037 / 0.053 | 0.027 |
| AIC | 22905.022 | 22578.22 | 22767.56 | 20764.118 | 20410.986 | 20492.061 |

Supplemental Table 5. Overall model results for tetracyclines resistance.

|  | Tetracyclines 2021 | | | Tetracyclines 2022 | | |
| --- | --- | --- | --- | --- | --- | --- |
| *Predictors* | Null | Full RI | Full no RI | Null | Full RI | Full no RI |
| (Intercept) | 0.1 (0.09 – 0.11) | 0.07 (0.05 – 0.09) | 0.07 (0.06 – 0.10) | 0.1 (0.09 – 0.11) | 0.06 (0.04 – 0.08) | 0.07 (0.05 – 0.09) |
| monthofyear [2] |  | 0.88 (0.71 – 1.10) | 0.89 (0.71 – 1.10) |  | 1.03 (0.82 – 1.28) | 1.05 (0.84 – 1.30) |
| monthofyear [3] |  | 1.03 (0.84 – 1.26) | 1.03 (0.84 – 1.26) |  | 1.07 (0.87 – 1.32) | 1.08 (0.87 – 1.33) |
| monthofyear [4] |  | 0.92 (0.75 – 1.14) | 0.93 (0.76 – 1.15) |  | 0.99 (0.79 – 1.23) | 0.99 (0.79 – 1.23) |
| monthofyear [5] |  | 0.89 (0.72 – 1.11) | 0.91 (0.73 – 1.13) |  | 1.07 (0.86 – 1.33) | 1.05 (0.85 – 1.31) |
| monthofyear [6] |  | 0.87 (0.70 – 1.07) | 0.87 (0.71 – 1.08) |  | 1.15 (0.93 – 1.43) | 1.15 (0.93 – 1.43) |
| monthofyear [7] |  | 0.98 (0.79 – 1.21) | 0.99 (0.80 – 1.22) |  | 1.03 (0.82 – 1.28) | 1.03 (0.83 – 1.28) |
| monthofyear [8] |  | 1.04 (0.84 – 1.29) | 1.04 (0.84 – 1.29) |  | 1.09 (0.88 – 1.35) | 1.09 (0.88 – 1.35) |
| monthofyear [9] |  | 0.86 (0.69 – 1.07) | 0.86 (0.69 – 1.07) |  | 1.12 (0.90 – 1.40) | 1.11 (0.90 – 1.39) |
| monthofyear [10] |  | 0.96 (0.78 – 1.20) | 0.97 (0.78 – 1.20) |  | 1.03 (0.82 – 1.29) | 1.02 (0.81 – 1.28) |
| monthofyear [11] |  | 0.96 (0.77 – 1.20) | 0.96 (0.77 – 1.19) |  | 1.17 (0.93 – 1.46) | 1.16 (0.93 – 1.45) |
| monthofyear [12] |  | 1.02 (0.82 – 1.26) | 1.01 (0.82 – 1.26) |  | 1.33 (1.07 – 1.66) | 1.33 (1.07 – 1.65) |
| Gender [M] |  | 1.22 (0.98 – 1.52) | 1.19 (0.96 – 1.49) |  | 1.16 (0.93 – 1.44) | 1.13 (0.92 – 1.42) |
| age_cat45-55 |  | 0.91 (0.72 – 1.14) | 0.9 (0.72 – 1.13) |  | 0.96 (0.76 – 1.21) | 0.94 (0.74 – 1.19) |
| age_cat55-65 |  | 0.82 (0.67 – 1.00) | 0.82 (0.67 – 1.00) |  | 0.98 (0.80 – 1.21) | 0.97 (0.79 – 1.19) |
| age_cat65-75 |  | 0.88 (0.73 – 1.07) | 0.87 (0.72 – 1.06) |  | 0.99 (0.81 – 1.21) | 0.97 (0.80 – 1.19) |
| age_cat75-85 |  | 0.97 (0.79 – 1.19) | 0.96 (0.78 – 1.18) |  | 1.08 (0.88 – 1.33) | 1.06 (0.86 – 1.30) |
| age cat [GT85] |  | 0.97 (0.76 – 1.23) | 0.96 (0.76 – 1.22) |  | 1.1 (0.86 – 1.42) | 1.07 (0.83 – 1.37) |
| Elix pvd |  | 1.43 (1.29 – 1.59) | 1.43 (1.29 – 1.58) |  | 1.27 (1.15 – 1.41) | 1.27 (1.14 – 1.40) |
| Elix parlysis |  | 1.27 (1.05 – 1.54) | 1.25 (1.03 – 1.50) |  | 1.22 (1.00 – 1.48) | 1.21 (1.00 – 1.46) |
| Elix neurological |  | 1.18 (1.05 – 1.34) | 1.18 (1.04 – 1.33) |  | 1.35 (1.20 – 1.52) | 1.34 (1.19 – 1.51) |
| Elix DM any |  | 1.21 (1.10 – 1.33) | 1.21 (1.09 – 1.33) |  | 1.21 (1.10 – 1.34) | 1.2 (1.09 – 1.33) |
| Elix HIVAIDS |  | 1.71 (1.23 – 2.38) | 1.78 (1.27 – 2.44) |  | 2.27 (1.67 – 3.08) | 2.37 (1.74 – 3.18) |
| Elix deficiencyanemia |  | 1.22 (1.09 – 1.37) | 1.23 (1.09 – 1.38) |  | 1.12 (1.00 – 1.26) | 1.13 (1.01 – 1.27) |
| Elix depression |  | 1.11 (1.00 – 1.22) | 1.1 (1.00 – 1.21) |  | 1.1 (1.00 – 1.21) | 1.08 (0.99 – 1.19) |
| Elix alcoholabuse |  | 0.85 (0.74 – 0.97) | 0.85 (0.74 – 0.97) |  | 1.16 (1.03 – 1.32) | 1.17 (1.03 – 1.32) |
| Tetracyclines D7 |  | 1.32 (0.21 – 8.37) | 1.45 (0.19 – 8.58) |  | 0.5 (0.09 – 2.78) | 0.51 (0.08 – 2.77) |
| Tetracyclines D30 |  | 15.37 (6.26 – 37.72) | 16.1 (6.80 – 41.06) |  | 8.08 (3.41 – 19.16) | 9.29 (4.02 – 22.39) |
| Tetracyclines D90 |  | 1.84 (0.85 – 3.96) | 1.88 (0.83 – 3.89) |  | 2.12 (1.04 – 4.33) | 2.15 (1.02 – 4.21) |
| *Random Effects* |  |  |  |  |  |  |
| σ2 | 3.29 | 3.29 |  | 3.29 | 3.29 |  |
| τ00 | 0.079 | 0.079 |  | 0.12 | 0.118 |  |
| ICC | 0.023 | 0.023 |  | 0.035 | 0.035 |  |
| Marginal R2 / Conditional R2 | 0.000 / 0.023 | 0.029 / 0.051 | 0.014 | 0.000 / 0.035 | 0.027 / 0.060 | 0.015 |
| AIC | 14083.349 | 13898.824 | 13943.683 | 14010.88 | 13834.414 | 13932.635 |

Supplemental Table 6. Overall model results for trimethoprim-sulfamethoxazole resistance.

|  | Trimethoprim-Sulfamethoxazole 2021 | | | Trimethoprim-Sulfamethoxazole 2022 | | |
| --- | --- | --- | --- | --- | --- | --- |
| *Predictors* | Null | Full RI | Full no RI | Null | Full RI | Full no RI |
| (Intercept) | 0.04 (0.03 – 0.04) | 0.02 (0.02 – 0.04) | 0.03 (0.02 – 0.04) | 0.04 (0.04 – 0.05) | 0.03 (0.02 – 0.04) | 0.03 (0.02 – 0.05) |
| monthofyear [2] |  | 1.32 (0.96 – 1.81) | 1.35 (0.98 – 1.85) |  | 1.31 (0.97 – 1.78) | 1.34 (0.99 – 1.81) |
| monthofyear [3] |  | 1.22 (0.90 – 1.66) | 1.23 (0.91 – 1.68) |  | 1.03 (0.76 – 1.41) | 1.03 (0.76 – 1.41) |
| monthofyear [4] |  | 1.12 (0.81 – 1.55) | 1.12 (0.82 – 1.55) |  | 1.35 (0.99 – 1.82) | 1.35 (1.00 – 1.83) |
| monthofyear [5] |  | 1.39 (1.02 – 1.90) | 1.39 (1.02 – 1.89) |  | 1.29 (0.95 – 1.75) | 1.26 (0.93 – 1.71) |
| monthofyear [6] |  | 1.49 (1.11 – 2.02) | 1.5 (1.11 – 2.02) |  | 1.2 (0.88 – 1.64) | 1.21 (0.89 – 1.65) |
| monthofyear [7] |  | 1.03 (0.74 – 1.44) | 1.02 (0.73 – 1.42) |  | 1.51 (1.12 – 2.03) | 1.49 (1.11 – 2.00) |
| monthofyear [8] |  | 1.36 (0.99 – 1.86) | 1.37 (1.01 – 1.88) |  | 1.19 (0.87 – 1.62) | 1.18 (0.87 – 1.60) |
| monthofyear [9] |  | 1.65 (1.22 – 2.24) | 1.66 (1.23 – 2.24) |  | 1.24 (0.91 – 1.70) | 1.22 (0.89 – 1.67) |
| monthofyear [10] |  | 1.19 (0.86 – 1.65) | 1.22 (0.89 – 1.69) |  | 1.1 (0.80 – 1.53) | 1.08 (0.78 – 1.50) |
| monthofyear [11] |  | 1.27 (0.91 – 1.76) | 1.27 (0.92 – 1.75) |  | 1.14 (0.82 – 1.58) | 1.14 (0.82 – 1.57) |
| monthofyear [12] |  | 1.29 (0.93 – 1.78) | 1.28 (0.93 – 1.77) |  | 1.62 (1.20 – 2.19) | 1.6 (1.19 – 2.17) |
| Gender [M] |  | 0.92 (0.70 – 1.22) | 0.9 (0.69 – 1.20) |  | 1.02 (0.77 – 1.36) | 1.02 (0.78 – 1.37) |
| age_cat45-55 |  | 1.1 (0.81 – 1.49) | 1.12 (0.83 – 1.52) |  | 1.15 (0.85 – 1.56) | 1.13 (0.83 – 1.53) |
| age_cat55-65 |  | 0.98 (0.75 – 1.29) | 0.99 (0.76 – 1.30) |  | 0.93 (0.71 – 1.22) | 0.96 (0.74 – 1.26) |
| age_cat65-75 |  | 0.98 (0.76 – 1.28) | 0.97 (0.75 – 1.26) |  | 0.94 (0.72 – 1.21) | 0.93 (0.72 – 1.20) |
| age_cat75-85 |  | 1.21 (0.91 – 1.59) | 1.17 (0.89 – 1.55) |  | 1.07 (0.82 – 1.40) | 1.06 (0.82 – 1.39) |
| age cat [GT85] |  | 1.42 (1.03 – 1.94) | 1.41 (1.03 – 1.93) |  | 1.12 (0.81 – 1.55) | 1.07 (0.78 – 1.49) |
| Elix pvd |  | 1.21 (1.04 – 1.40) | 1.17 (1.01 – 1.34) |  | 1.21 (1.05 – 1.39) | 1.18 (1.02 – 1.36) |
| Elix parlysis |  | 1.42 (1.11 – 1.83) | 1.43 (1.11 – 1.82) |  | 1.61 (1.27 – 2.04) | 1.66 (1.31 – 2.09) |
| Elix neurological |  | 1.24 (1.05 – 1.46) | 1.23 (1.04 – 1.45) |  | 1.21 (1.02 – 1.43) | 1.19 (1.01 – 1.40) |
| Elix HIVAIDS |  | 2.52 (1.73 – 3.68) | 2.68 (1.82 – 3.83) |  | 2.39 (1.65 – 3.47) | 2.65 (1.82 – 3.76) |
| Elix fluidelectrolyte |  | 1.27 (1.10 – 1.46) | 1.24 (1.08 – 1.42) |  | 1.16 (1.01 – 1.33) | 1.16 (1.01 – 1.32) |
| trim sulfa D7 |  | 1.73 (1.19 – 2.51) | 1.7 (1.16 – 2.42) |  | 1.62 (1.11 – 2.36) | 1.59 (1.08 – 2.28) |
| trim sulfa D30 |  | 2.23 (1.59 – 3.13) | 2.14 (1.52 – 2.96) |  | 1.95 (1.37 – 2.79) | 1.9 (1.32 – 2.67) |
| trim sulfa D90 |  | 1.73 (1.32 – 2.28) | 1.72 (1.30 – 2.24) |  | 1.79 (1.37 – 2.35) | 1.77 (1.35 – 2.30) |
| *Random Effects* |  |  |  |  |  |  |
| σ2 | 3.29 | 3.29 |  | 3.29 | 3.29 |  |
| τ00 | 0.306 | 0.318 |  | 0.275 | 0.271 |  |
| ICC | 0.085 | 0.088 |  | 0.077 | 0.076 |  |
| Marginal R2 / Conditional R2 | 0.000 / 0.085 | 0.033 / 0.119 | 0.009 | 0.000 / 0.077 | 0.028 / 0.102 | 0.009 |
| AIC | 8353.94 | 8240.368 | 8402.607 | 8416.848 | 8324.493 | 8466.336 |

Supplemental Table 7. Random intercept model results for clindamycin resistance, stratified by MRSA/MSSA.

|  | Clindamycin 2021 | | Clindamycin 2022 | |
| --- | --- | --- | --- | --- |
| *Predictors* | MRSA | MSSA | MRSA | MSSA |
| (Intercept) | 0.25 (0.18 – 0.36) | 0.23 (0.17 – 0.30) | 0.25 (0.18 – 0.36) | 0.21 (0.15 – 0.27) |
| monthofyear [2] | 0.94 (0.73 – 1.22) | 0.93 (0.74 – 1.15) | 0.97 (0.75 – 1.27) | 1.02 (0.82 – 1.26) |
| monthofyear [3] | 1.12 (0.87 – 1.43) | 0.99 (0.81 – 1.21) | 1.13 (0.88 – 1.46) | 1.01 (0.82 – 1.24) |
| monthofyear [4] | 0.84 (0.65 – 1.07) | 0.97 (0.79 – 1.19) | 0.84 (0.64 – 1.10) | 1.05 (0.85 – 1.30) |
| monthofyear [5] | 0.93 (0.73 – 1.20) | 1.08 (0.87 – 1.33) | 0.99 (0.76 – 1.29) | 0.96 (0.78 – 1.19) |
| monthofyear [6] | 0.81 (0.63 – 1.04) | 0.91 (0.74 – 1.12) | 1.17 (0.91 – 1.52) | 1.11 (0.90 – 1.37) |
| monthofyear [7] | 0.81 (0.63 – 1.04) | 0.8 (0.64 – 0.99) | 1 (0.78 – 1.29) | 0.8 (0.64 – 1.00) |
| monthofyear [8] | 0.85 (0.66 – 1.10) | 0.87 (0.71 – 1.08) | 1.07 (0.82 – 1.38) | 0.94 (0.76 – 1.16) |
| monthofyear [9] | 0.75 (0.58 – 0.97) | 0.9 (0.73 – 1.11) | 1.02 (0.78 – 1.33) | 0.99 (0.80 – 1.24) |
| monthofyear [10] | 0.73 (0.56 – 0.94) | 0.84 (0.68 – 1.05) | 1.03 (0.79 – 1.35) | 1.03 (0.82 – 1.28) |
| monthofyear [11] | 0.84 (0.64 – 1.09) | 0.79 (0.64 – 0.99) | 0.93 (0.71 – 1.21) | 1.09 (0.88 – 1.36) |
| monthofyear [12] | 0.67 (0.51 – 0.87) | 0.87 (0.70 – 1.08) | 1.06 (0.81 – 1.38) | 0.98 (0.78 – 1.23) |
| Gender [M] | 0.98 (0.77 – 1.27) | 0.93 (0.77 – 1.13) | 0.93 (0.72 – 1.19) | 1.02 (0.83 – 1.24) |
| age_cat45-55 | 1.11 (0.85 – 1.45) | 0.98 (0.79 – 1.20) | 1.06 (0.81 – 1.40) | 1.04 (0.84 – 1.28) |
| age_cat55-65 | 1.11 (0.88 – 1.39) | 0.97 (0.81 – 1.17) | 1.02 (0.80 – 1.30) | 0.94 (0.77 – 1.14) |
| age_cat65-75 | 1.38 (1.10 – 1.73) | 0.97 (0.81 – 1.16) | 1.13 (0.90 – 1.44) | 0.94 (0.78 – 1.14) |
| age_cat75-85 | 1.58 (1.24 – 2.02) | 1.1 (0.90 – 1.34) | 1.24 (0.97 – 1.60) | 0.97 (0.79 – 1.18) |
| age cat [GT85] | 1.95 (1.47 – 2.60) | 1.16 (0.90 – 1.48) | 1.71 (1.26 – 2.32) | 1.21 (0.94 – 1.55) |
| Clindamycin D7 | 2.12 (1.44 – 3.13) | 0.89 (0.57 – 1.40) | 1.93 (1.14 – 3.28) | 1.93 (1.31 – 2.83) |
| Clindamycin D30 | 7.04 (3.43 – 14.46) | 8.05 (4.68 – 13.85) | 8.99 (4.37 – 18.47) | 3.31 (2.02 – 5.42) |
| Clindamycin D90 | 2.47 (1.66 – 3.66) | 1.72 (1.21 – 2.44) | 2.64 (1.71 – 4.06) | 1.34 (0.93 – 1.95) |
| Macrolides D7 | 0.94 (0.72 – 1.23) | 1.28 (1.01 – 1.62) | 1.09 (0.84 – 1.40) | 1.14 (0.89 – 1.45) |
| Macrolides D30 | 1.99 (1.54 – 2.57) | 1.57 (1.23 – 1.99) | 2.06 (1.62 – 2.61) | 1.52 (1.20 – 1.93) |
| Macrolides D90 | 1.27 (1.06 – 1.54) | 1.55 (1.31 – 1.85) | 1.31 (1.08 – 1.58) | 1.56 (1.32 – 1.86) |
| Elix pvd | 1.44 (1.27 – 1.62) | 1.06 (0.95 – 1.18) | 1.29 (1.14 – 1.46) | 1.04 (0.94 – 1.16) |
| Elix parlysis | 1.73 (1.37 – 2.19) | 1.23 (0.95 – 1.58) | 1.55 (1.21 – 1.97) | 1.19 (0.92 – 1.54) |
| Elix neurological | 1.12 (0.97 – 1.29) | 1.12 (0.98 – 1.28) | 1.1 (0.95 – 1.27) | 1.12 (0.98 – 1.28) |
| Elix chronicpulmonary | 1.12 (1.00 – 1.26) | 1.16 (1.05 – 1.29) | 1.09 (0.97 – 1.24) | 1.1 (0.99 – 1.22) |
| Elix DM any | 1.05 (0.93 – 1.19) | 1.09 (0.99 – 1.21) | 1.07 (0.94 – 1.21) | 1.21 (1.09 – 1.33) |
| Elix renalfailure | 1.27 (1.11 – 1.44) | 1.03 (0.92 – 1.15) | 1.38 (1.21 – 1.57) | 1.02 (0.91 – 1.13) |
| Elix pepticulcer | 0.78 (0.50 – 1.21) | 0.76 (0.52 – 1.10) | 0.76 (0.50 – 1.16) | 0.68 (0.46 – 1.02) |
| Elix HIVAIDS | 1.34 (0.91 – 1.98) | 1.76 (1.21 – 2.55) | 1.44 (0.99 – 2.08) | 1.46 (0.98 – 2.19) |
| Elix lymphoma | 1.31 (0.88 – 1.95) | 1.18 (0.87 – 1.62) | 1.11 (0.72 – 1.70) | 1.44 (1.07 – 1.93) |
| Elix obesity | 0.98 (0.87 – 1.11) | 0.95 (0.86 – 1.05) | 1.01 (0.90 – 1.15) | 0.87 (0.79 – 0.96) |
| Elix deficiencyanemia | 1.34 (1.17 – 1.54) | 0.97 (0.85 – 1.10) | 1.21 (1.05 – 1.39) | 1.11 (0.98 – 1.25) |
| Elix psychoses | 1.28 (1.00 – 1.63) | 1.07 (0.84 – 1.35) | 1.16 (0.91 – 1.49) | 1.22 (0.97 – 1.54) |
| Elix depression | 1.11 (0.99 – 1.24) | 1.15 (1.05 – 1.27) | 1.14 (1.01 – 1.28) | 1.12 (1.02 – 1.23) |
| *Random Effects* |  |  |  |  |
| σ^2^ | 3.29 | 3.29 | 3.29 | 3.29 |
| τ_00_ | 0.158 | 0.149 | 0.125 | 0.109 |
| ICC | 0.046 | 0.043 | 0.037 | 0.032 |
| Marginal R^2^ / Conditional R^2^ | 0.088 / 0.130 | 0.029 / 0.071 | 0.075 / 0.109 | 0.027 / 0.058 |
| AIC | 8275.046 | 12543.993 | 7800.493 | 12076.942 |

Supplemental Table 8. Random intercept model results for macrolides resistance, stratified by MRSA/MSSA.

|  | Macrolides 2021 | | Macrolides 2022 | |
| --- | --- | --- | --- | --- |
| *Predictors* | MRSA | MSSA | MRSA | MSSA |
| (Intercept) | 4.86 (3.09 – 7.62) | 0.53 (0.41 – 0.68) | 5.1 (3.21 – 8.11) | 0.48 (0.37 – 0.63) |
| monthofyear [2] | 0.88 (0.62 – 1.26) | 0.92 (0.75 – 1.12) | 1.26 (0.88 – 1.82) | 0.95 (0.77 – 1.17) |
| monthofyear [3] | 0.93 (0.66 – 1.30) | 1.04 (0.87 – 1.26) | 1.04 (0.74 – 1.45) | 1.02 (0.83 – 1.24) |
| monthofyear [4] | 0.86 (0.62 – 1.21) | 1.13 (0.94 – 1.37) | 0.96 (0.68 – 1.35) | 1.09 (0.89 – 1.34) |
| monthofyear [5] | 0.94 (0.67 – 1.32) | 0.96 (0.79 – 1.17) | 0.96 (0.68 – 1.34) | 0.99 (0.81 – 1.22) |
| monthofyear [6] | 0.87 (0.62 – 1.23) | 0.98 (0.81 – 1.18) | 1.02 (0.72 – 1.45) | 0.96 (0.78 – 1.18) |
| monthofyear [7] | 0.93 (0.66 – 1.31) | 0.98 (0.80 – 1.19) | 1.03 (0.73 – 1.44) | 0.86 (0.70 – 1.06) |
| monthofyear [8] | 0.93 (0.65 – 1.31) | 0.99 (0.81 – 1.20) | 0.94 (0.66 – 1.32) | 0.94 (0.77 – 1.15) |
| monthofyear [9] | 0.95 (0.67 – 1.36) | 1.06 (0.87 – 1.29) | 0.93 (0.66 – 1.30) | 0.92 (0.75 – 1.14) |
| monthofyear [10] | 0.89 (0.63 – 1.27) | 0.97 (0.79 – 1.18) | 1.01 (0.71 – 1.45) | 1 (0.81 – 1.23) |
| monthofyear [11] | 0.78 (0.55 – 1.11) | 0.87 (0.71 – 1.06) | 0.94 (0.67 – 1.33) | 1 (0.81 – 1.24) |
| monthofyear [12] | 0.88 (0.62 – 1.26) | 0.99 (0.81 – 1.21) | 0.99 (0.69 – 1.40) | 1.06 (0.86 – 1.32) |
| Gender [M] | 0.92 (0.67 – 1.27) | 0.88 (0.74 – 1.05) | 0.86 (0.62 – 1.20) | 0.94 (0.78 – 1.13) |
| age_cat45-55 | 1.19 (0.86 – 1.65) | 1.02 (0.84 – 1.23) | 0.96 (0.67 – 1.37) | 1.09 (0.89 – 1.33) |
| age_cat55-65 | 0.99 (0.75 – 1.31) | 1.08 (0.92 – 1.28) | 0.93 (0.68 – 1.27) | 0.94 (0.79 – 1.13) |
| age_cat65-75 | 1.11 (0.85 – 1.45) | 1.03 (0.87 – 1.20) | 0.87 (0.65 – 1.17) | 1.03 (0.87 – 1.23) |
| age_cat75-85 | 1.08 (0.80 – 1.46) | 1.13 (0.95 – 1.35) | 0.95 (0.69 – 1.31) | 1.05 (0.87 – 1.26) |
| age cat [GT85] | 1.54 (1.04 – 2.26) | 1.31 (1.05 – 1.63) | 1.25 (0.82 – 1.91) | 1.44 (1.14 – 1.82) |
| Clindamycin D7 | 1.26 (0.68 – 2.34) | 1.19 (0.80 – 1.77) | 2.81 (0.99 – 7.95) | 1.5 (0.99 – 2.28) |
| Clindamycin D30 | 4.74 (1.13 – 19.97) | 2.61 (1.54 – 4.43) | 1.56 (0.59 – 4.09) | 2.91 (1.66 – 5.11) |
| Clindamycin D90 | 1.11 (0.62 – 1.97) | 1.22 (0.86 – 1.73) | 0.98 (0.53 – 1.81) | 1.33 (0.90 – 1.96) |
| Macrolides D7 | 0.83 (0.58 – 1.19) | 1.29 (1.02 – 1.63) | 1.23 (0.84 – 1.80) | 1.09 (0.86 – 1.39) |
| Macrolides D30 | 1.67 (1.08 – 2.60) | 1.43 (1.13 – 1.83) | 1.84 (1.21 – 2.79) | 1.62 (1.26 – 2.08) |
| Macrolides D90 | 1.34 (1.00 – 1.78) | 1.53 (1.29 – 1.82) | 1.27 (0.96 – 1.69) | 1.5 (1.26 – 1.79) |
| Elix parlysis | 1.07 (0.76 – 1.51) | 1.22 (0.96 – 1.56) | 1.25 (0.88 – 1.80) | 1.06 (0.82 – 1.38) |
| Elix neurological | 1.04 (0.85 – 1.27) | 1.05 (0.93 – 1.19) | 1.01 (0.83 – 1.24) | 1.08 (0.95 – 1.23) |
| Elix chronicpulmonary | 1.18 (1.00 – 1.38) | 1.16 (1.06 – 1.27) | 1.23 (1.05 – 1.45) | 1.21 (1.09 – 1.33) |
| Elix HIVAIDS | 2.98 (1.37 – 6.46) | 1.37 (0.93 – 2.01) | 2.05 (1.09 – 3.87) | 1.75 (1.12 – 2.72) |
| Elix obesity | 0.94 (0.81 – 1.10) | 0.92 (0.84 – 1.00) | 0.93 (0.80 – 1.09) | 0.88 (0.80 – 0.96) |
| Elix drugabuse | 1.25 (1.01 – 1.56) | 1.2 (1.05 – 1.36) | 1.28 (1.03 – 1.60) | 1.26 (1.09 – 1.45) |
| Elix psychoses | 1.26 (0.88 – 1.83) | 1.04 (0.83 – 1.30) | 1.16 (0.81 – 1.66) | 1.19 (0.95 – 1.49) |
| Elix depression | 1.23 (1.05 – 1.43) | 1.04 (0.95 – 1.14) | 1.01 (0.87 – 1.18) | 1.03 (0.94 – 1.13) |
| *Random Effects* |  |  |  |  |
| σ^2^ | 3.29 | 3.29 | 3.29 | 3.29 |
| τ_00_ | 0.043 | 0.074 | 0.092 | 0.05 |
| ICC | 0.013 | 0.022 | 0.027 | 0.015 |
| Marginal R^2^ / Conditional R^2^ | 0.037 / 0.049 | 0.017 / 0.039 | 0.032 / 0.059 | 0.023 / 0.038 |
| AIC | 5234.667 | 13505.081 | 5019.236 | 12013.688 |

Supplemental Table 8. Random intercept model results for tetracyclines resistance, stratified by MRSA/MSSA.

|  | Tetracyclines 2021 | | Tetracyclines 2022 | |
| --- | --- | --- | --- | --- |
| *Predictors* | MRSA | MSSA | MRSA | MSSA |
| (Intercept) | 0.1 (0.06 – 0.15) | 0.06 (0.04 – 0.09) | 0.11 (0.07 – 0.16) | 0.04 (0.02 – 0.06) |
| monthofyear [2] | 0.89 (0.66 – 1.20) | 0.81 (0.57 – 1.14) | 0.94 (0.70 – 1.28) | 1.16 (0.83 – 1.64) |
| monthofyear [3] | 0.96 (0.72 – 1.29) | 1.08 (0.80 – 1.46) | 0.94 (0.70 – 1.26) | 1.3 (0.94 – 1.80) |
| monthofyear [4] | 0.81 (0.60 – 1.09) | 0.99 (0.73 – 1.36) | 0.87 (0.64 – 1.18) | 1.18 (0.84 – 1.66) |
| monthofyear [5] | 0.85 (0.63 – 1.15) | 0.9 (0.65 – 1.24) | 1.12 (0.83 – 1.50) | 1.12 (0.80 – 1.57) |
| monthofyear [6] | 0.8 (0.59 – 1.08) | 0.9 (0.66 – 1.23) | 1.23 (0.92 – 1.64) | 1.1 (0.78 – 1.56) |
| monthofyear [7] | 0.85 (0.63 – 1.16) | 1.08 (0.79 – 1.48) | 0.88 (0.65 – 1.19) | 1.3 (0.93 – 1.82) |
| monthofyear [8] | 1 (0.74 – 1.34) | 1.06 (0.78 – 1.45) | 1.06 (0.79 – 1.42) | 1.26 (0.90 – 1.75) |
| monthofyear [9] | 0.9 (0.67 – 1.22) | 0.76 (0.54 – 1.07) | 1.01 (0.75 – 1.37) | 1.32 (0.94 – 1.85) |
| monthofyear [10] | 0.93 (0.69 – 1.26) | 0.97 (0.70 – 1.34) | 0.94 (0.69 – 1.29) | 1.2 (0.85 – 1.70) |
| monthofyear [11] | 0.79 (0.57 – 1.09) | 1.12 (0.81 – 1.53) | 1.08 (0.80 – 1.47) | 1.27 (0.90 – 1.80) |
| monthofyear [12] | 1 (0.74 – 1.35) | 0.94 (0.68 – 1.31) | 1.29 (0.96 – 1.73) | 1.38 (0.98 – 1.95) |
| Gender [M] | 1.35 (0.96 – 1.89) | 1.06 (0.78 – 1.44) | 1.09 (0.80 – 1.48) | 1.1 (0.80 – 1.51) |
| age_cat45-55 | 0.89 (0.64 – 1.24) | 0.98 (0.71 – 1.35) | 0.93 (0.67 – 1.29) | 1.04 (0.72 – 1.49) |
| age_cat55-65 | 0.85 (0.64 – 1.13) | 0.85 (0.63 – 1.13) | 0.84 (0.63 – 1.11) | 1.17 (0.86 – 1.60) |
| age_cat65-75 | 0.88 (0.67 – 1.15) | 0.94 (0.71 – 1.24) | 0.88 (0.67 – 1.15) | 1.15 (0.85 – 1.55) |
| age_cat75-85 | 0.98 (0.73 – 1.31) | 0.97 (0.72 – 1.30) | 1.01 (0.76 – 1.35) | 1.16 (0.84 – 1.60) |
| age cat [GT85] | 0.97 (0.69 – 1.34) | 0.92 (0.64 – 1.33) | 1.06 (0.76 – 1.47) | 1 (0.67 – 1.49) |
| Tetracyclines D7 | 0.5 (0.02 – 14.74) | 2.8 (0.29 – 27.36) | 0.11 (0.01 – 1.09) | 0 (0.00 – Inf) |
| Tetracyclines D30 | 24.25 (5.94 – 98.93) | 11.27 (3.16 – 40.16) | 7.08 (2.01 – 24.93) | 149675221.03 (0.00 – Inf) |
| Tetracyclines D90 | 1.57 (0.50 – 4.89) | 2.25 (0.77 – 6.61) | 5.36 (2.24 – 12.86) | 0 (0.00 – Inf) |
| Elix pvd | 1.61 (1.40 – 1.86) | 1.24 (1.06 – 1.44) | 1.33 (1.15 – 1.53) | 1.16 (0.99 – 1.35) |
| Elix parlysis | 1.12 (0.88 – 1.44) | 1.22 (0.88 – 1.69) | 1.12 (0.87 – 1.43) | 1.02 (0.71 – 1.45) |
| Elix neurological | 1.18 (1.00 – 1.39) | 1.01 (0.83 – 1.24) | 1.29 (1.10 – 1.51) | 1.23 (1.02 – 1.49) |
| Elix DM any | 1.21 (1.05 – 1.39) | 1.21 (1.05 – 1.39) | 1.17 (1.02 – 1.34) | 1.27 (1.10 – 1.47) |
| Elix HIVAIDS | 1.4 (0.90 – 2.18) | 1.85 (1.10 – 3.10) | 1.7 (1.14 – 2.53) | 2.38 (1.42 – 3.97) |
| Elix deficiencyanemia | 1.28 (1.09 – 1.50) | 1.08 (0.90 – 1.30) | 1.12 (0.96 – 1.31) | 1.01 (0.84 – 1.21) |
| Elix alcoholabuse | 0.75 (0.61 – 0.91) | 0.97 (0.80 – 1.18) | 1.17 (0.99 – 1.39) | 1.11 (0.92 – 1.35) |
| Elix depression | 1.02 (0.89 – 1.16) | 1.14 (0.98 – 1.31) | 1.09 (0.95 – 1.24) | 1.04 (0.90 – 1.20) |
| *Random Effects* |  |  |  |  |
| σ^2^ | 3.29 | 3.29 | 3.29 | 3.29 |
| τ_00_ | 0.167 | 0.06 | 0.242 | 0.063 |
| ICC | 0.048 | 0.018 | 0.068 | 0.019 |
| Marginal R^2^ / Conditional R^2^ | 0.042 / 0.089 | 0.017 / 0.035 | 0.031 / 0.098 | 0.118 / 0.135 |
| AIC | 6609.036 | 6902.736 | 6688.213 | 6685.656 |

Supplemental Table 9. Random intercept model results for trimethoprim-sulfamethoxazole resistance, stratified by MRSA/MSSA.

|  | Trimethoprim-Sulfamethoxazole 2021 | | Trimethoprim-Sulfamethoxazole 2022 | | |
| --- | --- | --- | --- | --- | --- |
| *Predictors* | MRSA | MSSA | MRSA | MSSA |  |
| (Intercept) | 0.06 (0.03 – 0.09) | 0.01 (0.01 – 0.03) | 0.06 (0.04 – 0.10) | 0.01 (0.01 – 0.02) |  |
| monthofyear [2] | 1.44 (0.99 – 2.08) | 0.85 (0.43 – 1.70) | 1.67 (1.16 – 2.42) | 0.69 (0.37 – 1.29) |  |
| monthofyear [3] | 1.17 (0.80 – 1.70) | 1.37 (0.77 – 2.44) | 1.25 (0.86 – 1.83) | 0.76 (0.42 – 1.36) |  |
| monthofyear [4] | 1.09 (0.75 – 1.60) | 0.99 (0.53 – 1.88) | 1.6 (1.10 – 2.33) | 0.98 (0.56 – 1.72) |  |
| monthofyear [5] | 1.4 (0.97 – 2.03) | 1.06 (0.56 – 2.01) | 1.41 (0.97 – 2.07) | 1.25 (0.74 – 2.12) |  |
| monthofyear [6] | 1.37 (0.95 – 1.99) | 1.78 (1.02 – 3.12) | 1.38 (0.94 – 2.02) | 0.95 (0.53 – 1.69) |  |
| monthofyear [7] | 0.91 (0.61 – 1.37) | 1.19 (0.64 – 2.21) | 1.84 (1.28 – 2.65) | 1.04 (0.60 – 1.82) |  |
| monthofyear [8] | 1.28 (0.88 – 1.88) | 1.33 (0.73 – 2.44) | 1.16 (0.78 – 1.73) | 1.4 (0.83 – 2.35) |  |
| monthofyear [9] | 1.73 (1.21 – 2.49) | 1.27 (0.69 – 2.34) | 1.42 (0.96 – 2.10) | 0.97 (0.54 – 1.73) |  |
| monthofyear [10] | 1.06 (0.71 – 1.57) | 1.38 (0.76 – 2.53) | 1.28 (0.86 – 1.90) | 0.85 (0.46 – 1.57) |  |
| monthofyear [11] | 1.13 (0.75 – 1.69) | 1.57 (0.87 – 2.85) | 1.3 (0.87 – 1.94) | 0.8 (0.43 – 1.48) |  |
| monthofyear [12] | 1.38 (0.94 – 2.01) | 0.75 (0.36 – 1.54) | 1.93 (1.33 – 2.80) | 1.13 (0.64 – 2.00) |  |
| Gender [M] | 0.72 (0.52 – 1.01) | 1.2 (0.70 – 2.08) | 0.88 (0.62 – 1.23) | 1.29 (0.73 – 2.26) |  |
| age_cat45-55 | 1.22 (0.83 – 1.79) | 0.95 (0.56 – 1.60) | 1.23 (0.85 – 1.79) | 1.24 (0.70 – 2.19) |  |
| age_cat55-65 | 1.22 (0.86 – 1.71) | 0.67 (0.41 – 1.08) | 0.92 (0.66 – 1.28) | 1.1 (0.66 – 1.83) |  |
| age_cat65-75 | 1.2 (0.86 – 1.67) | 0.76 (0.49 – 1.18) | 0.98 (0.71 – 1.34) | 0.92 (0.56 – 1.50) |  |
| age_cat75-85 | 1.43 (1.01 – 2.03) | 0.81 (0.50 – 1.33) | 1.03 (0.74 – 1.44) | 1.22 (0.74 – 2.03) |  |
| age cat [GT85] | 1.58 (1.07 – 2.33) | 0.95 (0.53 – 1.73) | 1.22 (0.83 – 1.80) | 0.74 (0.36 – 1.50) |  |
| trim sulfa D7 | 1.29 (0.83 – 2.03) | 2.44 (1.20 – 4.94) | 1.29 (0.81 – 2.07) | 1.69 (0.84 – 3.40) |  |
| trim sulfa D30 | 2.51 (1.69 – 3.73) | 1.54 (0.71 – 3.35) | 1.33 (0.84 – 2.11) | 4.01 (2.17 – 7.40) |  |
| trim sulfa D90 | 1.62 (1.18 – 2.22) | 1.43 (0.76 – 2.70) | 1.81 (1.33 – 2.47) | 1.06 (0.55 – 2.04) |  |
| Elix pvd | 1.33 (1.12 – 1.59) | 0.84 (0.61 – 1.15) | 1.19 (1.01 – 1.42) | 0.99 (0.74 – 1.33) |  |
| Elix parlysis | 1.16 (0.87 – 1.55) | 1.08 (0.56 – 2.06) | 1.29 (0.98 – 1.71) | 1.55 (0.91 – 2.63) |  |
| Elix neurological | 1.18 (0.98 – 1.44) | 0.89 (0.60 – 1.32) | 1.06 (0.88 – 1.29) | 1.11 (0.78 – 1.57) |  |
| Elix HIVAIDS | 2.42 (1.55 – 3.76) | 2.05 (0.87 – 4.80) | 2.11 (1.38 – 3.23) | 1.24 (0.44 – 3.50) |  |
| Elix fluidelectrolyte | 1.16 (0.98 – 1.37) | 1 (0.74 – 1.35) | 1.14 (0.96 – 1.35) | 0.89 (0.67 – 1.19) |  |
| *Random Effects* |  |  |  |  |  |
| σ^2^ | 3.29 | 3.29 | 3.29 | 3.29 |  |
| τ_00_ | 0.258 | 0.476 | 0.281 | 0.484 |  |
| ICC | 0.073 | 0.126 | 0.079 | 0.128 |  |
| Marginal R^2^ / Conditional R^2^ | 0.038 / 0.108 | 0.029 / 0.152 | 0.027 / 0.103 | 0.030 / 0.154 |  |
| AIC | 5010.365 | 2637.073 | 4991.458 | 2706.719 |  |
